# Hypergravity and microgravity exhibited reversal effects on the bone and muscle mass in mice

**DOI:** 10.1038/s41598-019-42829-z

**Published:** 2019-04-29

**Authors:** Tsukasa Tominari, Ryota Ichimaru, Keita Taniguchi, Akane Yumoto, Masaki Shirakawa, Chiho Matsumoto, Kenta Watanabe, Michiko Hirata, Yoshifumi Itoh, Dai Shiba, Chisato Miyaura, Masaki Inada

**Affiliations:** 1 Department of Biotechnology and Life Science, Tokyo University of Agriculture and Technology, 2-24-16 Nakacho, Koganei, Tokyo 184-8588 Japan; 2 JEM Utilization Center, Human Spaceflight Technology Directorate, JAXA, 2-1-1 Sengen, Tsukuba, Ibaraki 305-8505 Japan; 3 Institute of Global Innovation Research, Tokyo University of Agriculture and Technology, 2-24-16 Nakacho, Koganei, Tokyo 184-8588 Japan; 4 Kennedy Institute of Rheumatology, Nuffield Department of Orthopaedics, Rheumatology and Musculoskeletal Sciences, University of Oxford, Oxford, OX3 7FY UK

**Keywords:** Molecular biology, Diseases

## Abstract

Spaceflight is known to induce severe systemic bone loss and muscle atrophy of astronauts due to the circumstances of microgravity. We examined the influence of artificially produced 2G hypergravity on mice for bone and muscle mass with newly developed centrifuge device. We also analyzed the effects of microgravity (mostly 0G) and artificial produced 1G in ISS (international space station) on mouse bone mass. Experiment on the ground, the bone mass of humerus, femur and tibia was measured using micro-computed tomography (μCT), and the all bone mass was significantly increased in 2G compared with 1G control. In tibial bone, the mRNA expression of bone formation related genes such as *Osx* and *Bmp2* was elevated. The volume of triceps surae muscle was also increased in 2G compared with 1G control, and the mRNA expression of myogenic factors such as *Myod* and *Myh1* was elevated by 2G. On the other hand, microgravity in ISS significantly induced the loss of bone mass on humerus and tibia, compared with artificial 1G induced by centrifugation. Here, we firstly report that bone and muscle mass are regulated by the gravity with loaded force in both of positive and negative on the ground and in the space.

## Introduction

Long-term exposure of microgravity (μG) by spaceflight and bed rest on the ground result in severe osteoporosis and muscle atrophy whereas mechanical loading by exercise recover the bone and muscle mass^1,2^. Thus, mechanical force plays a critical role in bone and muscle metabolism. Since it has been limited opportunity to transport the cells and animals to the space for the experimental microgravity due to the high cost, the physiological study of microgravity is still limited. As the rodent model of mechanical unloading, hindlimb unloading, also called as tail suspension, sciatic denervation and cast immobilization, have been used for mimicking μG-induced osteoporosis and muscle atrophy^3–5^. On the other hand, the centrifugation system on the ground is useful model for the experimental hypergravity. There are various types of the centrifuge devices which are the tabletop centrifugers (such as 1-foot diameter centrifuge device) connected with a tissue culture incubator^6^, the large diameter centrifuge system at the European Space Agency (ESA)^7^ and a custom-made gondora-type centrifugal device with 1.5 m long-arm^8^. In the present study, we investigated the alteration of skeletal tissues on mice using newly developed a gondola-type centrifugal device with a 1.0 m arm attached mice cages.

Bone metabolism is precisely maintained by the balance between osteoclastic bone resorption and new bone formation by osteoblasts^9^. Osteoclast is a multi-nuclear giant cell differentiated from macrophage lineage cells and possesses the bone-resorbing activity, while osteoblast controls bone formation and mineralization. There are various reports of *in vitro* studies in osteoblasts and osteoclasts using excess 10G hypergravity^6,10–18^, however, excess hypergravity may be unsuitable for *in vivo* experiments due to some trouble such as vestibular dysfunction and compressional organ injury^19^.

In muscle, myogenic differentiation is regulated by various factors such as MyoD, myogenin, myogenic regulatory factor (MRF) 4 and myogenic factor (MYF) 5, and mature myotube express myosin heavy chains (MHCs)^20,21^. It is known that insulin-like growth factor (IGF)-1 stimulates myogenesis via AKT-mTOR1-mediated protein synthesis, while protein degradation by autophagy and ubiquitin-proteasome system negatively regulate myogenesis^22,23^. Hypergravity of 5–20G stimulated the proliferation and differentiation of C2C12 myoblastic cell line *in vitro* in a gravity-dependent manner^7^. Therefore, hypergravity may stimulate myogenesis and induce muscle hypertrophy.

Recently, the experimental circumstances for the microgravity have been quickly advanced by the utilization of space station. The Japan Aerospace Exploration Agency (JAXA) established a novel mouse cages for the space experiments^24,25^. Various experiments for microgravity have been performed at the Japanese Experimental Module, called as ‘Kibo’, in the International Space Station (ISS). In 2016, the 1st mouse project in space using mouse habitat unit (MHU) was conducted by JAXA. This project (called as MHU-1) used the Centrifuge-equipped Biological Experiment Facility (CBEF) and provided both microgravity (μG) and artificial 1G in space, and reported that artificial 1G in space mostly cancelled the bone loss and muscle atrophy due to μG^25^. In 2017, the 2nd mouse space project (called as MHU-2) was performed, and we analyzed bone mass in the detail in the space.

In this study, we compared the effects of artificially produced 2G hypergravity and 1G control on bone and muscle mass on the ground using a new gondola-type centrifugal device with a 1.0 m arm. We also compared the bone mass in artificial 1G using centrifuge to that in μG (almost 0G) in the space (or in ISS).

## Results

### The influences of 2G hypergravity in body weight, food intake and water intake

**Figure 1:**
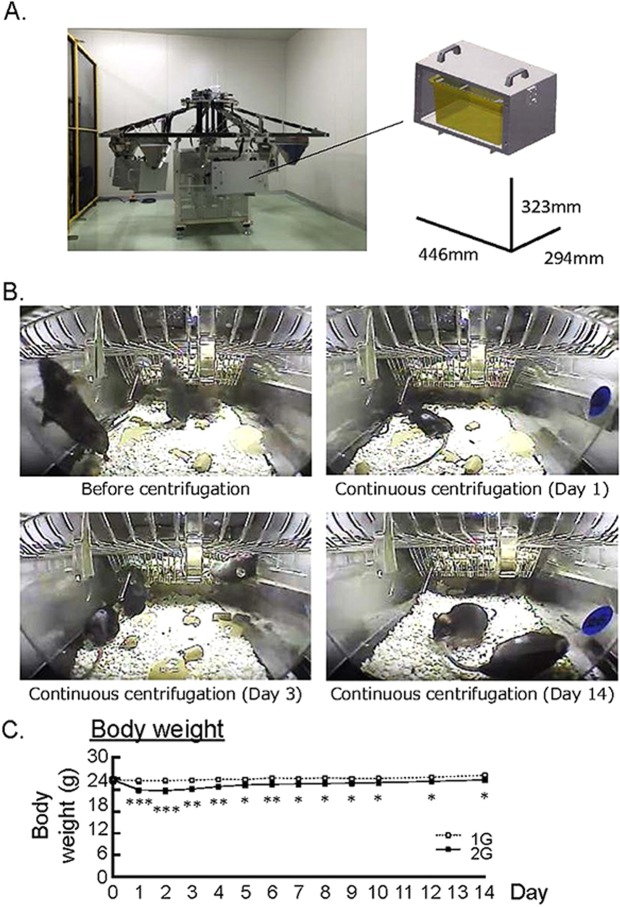
Experimental designs for 2G hypergravity and habitation data in mice. (**A**) The images showed a newly developed gondola-type centrifuge with a 1.0 m arm. (**B**) The video imaging on before continuous centrifugation of cages, day 1, day 3 and day 14 under the centrifugation. Mice walked in the cage freely before centrifugation on day 0. Mice moved slowly and lay face down on day 1. On day 3, mice gradually adjusted to 2G circumstance and walked in the cage. Mice finally walked freely in the cage on day 14. (**C**) The changes of body weight in 2G hypergravity experiment. Data are expressed as the mean ± SEM of 7–8 mice. A significant difference between the two groups was indicated; *P < *0*.*05*, **P < *0*.*01* and ***P < *0*.*001* vs 1G.

Mice were bred for 14 days in 2G hypergravity or 1G control using a new gondola-type centrifugal device (Fig. 1A). Figure 1B showed the live video imaging of mouse cage in the centrifuge device. Mice walked in the cage freely before centrifugation on day 0 (Fig. 1B, upper left panel). Although mice moved slowly and lay face down on day 1 after centrifugation (Fig. 1B, upper right panel), mice gradually adapted to the 2G hypergravity on day 3 (Fig. 1B, lower left panel). Finally, mice walked freely against the 2G in the cage on day 14 (Fig. 1B, lower right panel). In 2G group, body weight decreased on day 1 and recovered to about 96% of 1G control group, that was slightly lower than 1G (Fig. 1C). The intake of food and water were monitored using two mice of each group. Both dietary and water intakes were markedly reduced on day 1–2 in 2G group, but recovered to the similar level of 1G control group day by day. Therefore, the decrease in body weight may be due to the reduction of dietary and water intake on day 1–2 until the adaptation to 2G circumstance.

### 2G hypergravity increased the volume of triceps surae muscle

**Figure 2:**
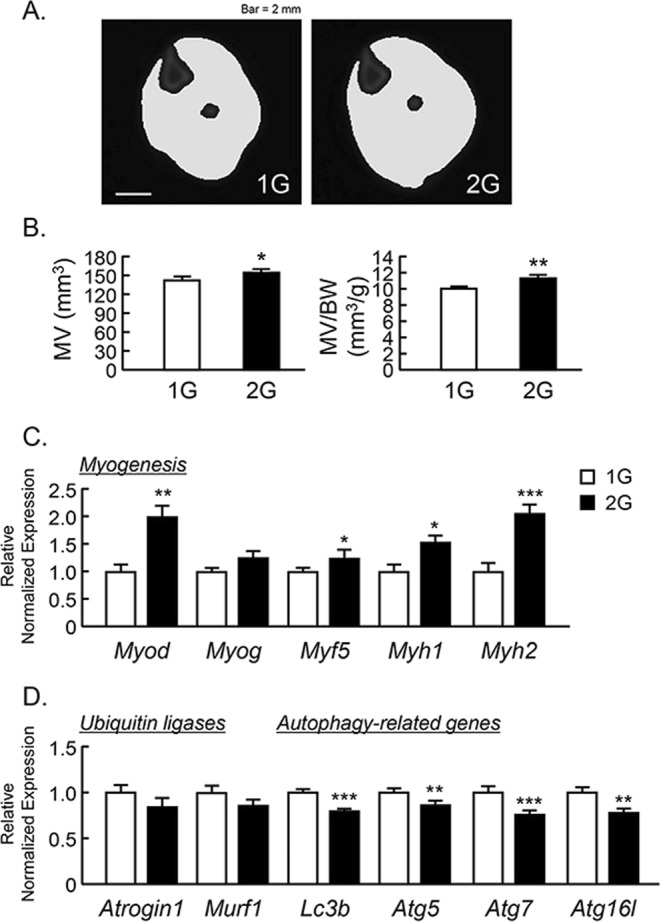
Analysis of triceps surae muscle volume using μCT in 2G mice. (**A**) The cross-sectional images of lower leg at center rejoin with muscle, were reconstructed by μCT. (**B**) The triceps surae muscle volume (MV) and MV per body weight (BW) were analyzed by μCT. (**C**,**D**) The mRNA expression of myogenic genes (*Myod*, *Myog*, *Myf5*, *Myh1* and *Myh2*), muscle-specific ubiquitin ligases (*Atrogin1* and *Murf1*) and autophagy-related genes (*Lc3b*, *Atg5*, *Atg7* and *Atg16L*) in quadriceps were measured using qPCR. In qPCR analysis, all genes were normalized by three reference genes (*Actb*, 18 *s* and *Hprt*). Data are expressed as the mean ± SEM of 7–8 mice. A significant difference between the two groups was indicated; *P < *0*.*05*, **P < *0*.*01* and ***P < *0*.*001* vs 1G.

**Figure 3:**
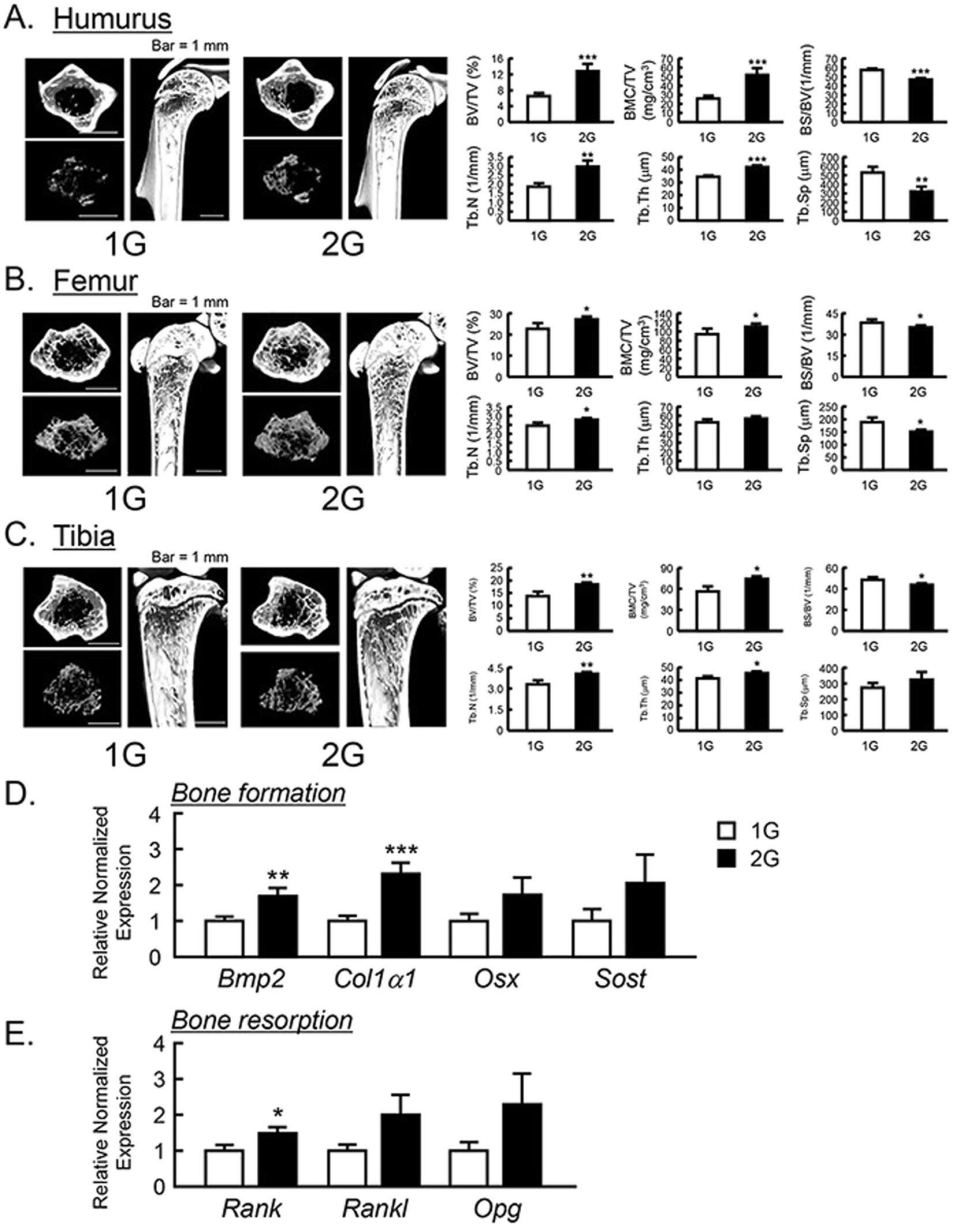
The μCT analysis for the bone mass of humurus, femur and tibia in 2G mice. The three-dimensional images (horizontal section, isolated trabecular bone of horizontal section, and vertical section) were shown in upper panels, and trabecular bone mass parameters were analyzed by μCT and shown in lower panels in humurus at the proximal region (**A**), femur at the distal region (**B**) and tibia at the proximal region (**C**). The μCT images showed representative bones of 7–8 mice. The mRNA expression of bone formation-related genes (**D**) and osteoclastogenesis-related genes (**E**) were measured using qPCR in tibial trabecular bone with bone marrows. In qPCR analysis, all genes were normalized by three reference genes (*Actb*, 18 *s* and *Hprt*). Data are expressed as the mean ± SEM of 7–8 mice. A significant difference between the two groups was indicated; *P < *0*.*05*, **P < *0*.*01* and ***P < *0*.*001* vs 1G.

To examine the effects of 2G hypergravity on muscle volume, we measured the volume of the triceps surae muscle in center region of lower leg using μCT. The muscle images of cross sectional area were shown in Fig. 2A. 2G enhanced the muscle volume and muscle volume per body weight (Fig. 2B). Using musculus quadriceps, we analyzed the mRNA expression of muscle related genes using RT-qPCR. 2G hypergravity enhanced the mRNA expression of myogenic genes, *Myod*, *Myf5*, *Myh1* and *Myh2*, in mouse muscles (Fig. 2C). In contrast, 2G hypergravity tended to downregulate the expression of ubiquitin ligases (*Atrogin1* and *Murf1*) and significantly suppressed the expression of autophagy related genes (*Lc3b*, *Atg5*, *Atg7* and *Atg16l*) in quadriceps (Fig. 3D). Therefore, muscle hypertrophy in 2G group may be due to both the increase in myogenic genes expression and the suppression of degradation genes in muscle.

### The changes in bone mass by 2G hypergravity in mice

To examine the influences of 2G hypergravity on bone mass, we analyzed the bone volume of humerus, femur, tibia and calvaria using μCT. BV/TV, BMC/TV, Tb.N, Tb.Th, BS/BV and Tb.Sp were calculated as the parameters of trabecular bone mass. The three-dimensional μCT images of humerus at the proximal area, femur at the distal area and tibia at the proximal area were taken. The reconstructed μCT images indicated that the trabecular bone mass in these areas was clearly increased in 2G-loaded mice (Fig. 3A–C, upper images) compared to 1G control. All of BV/TV, BMC/TV, Tb.N and Tb.Th of humerus, femur and tibia were clearly elevated, while BS/BV and Tb.Sp were suppressed by 2G (Fig. 3A–C, lower graphs). These data indicate that 2G hypergravity enhanced the bone mass in humurs, femur and tibia.

To determine the mechanism for bone mass increased by 2G, the mRNA expression of bone formative and resorptive genes in tibia was measured using RT-qPCR. In trabecular bone with bone marrow, 2G enhanced the mRNA expression of all of bone formation marker genes, bone morphogenetic protein-2 (*Bmp2*) and collagen type 1 α1 (*Col1a1*), osterix (*Osx*) and sclerostin (*Sost*) but the expression of *Osx* and *Sost* was not significantly changed (Fig. 3D). In the expression of osteoclastogenesis-related genes, receptor activator of NFκB (*Rank*) was elevated and other gene such as RANK ligand (*Rankl*) and osteoprotegerin (*Opg*) was tended to elevated, but not significant (Fig. 3E). These data indicated that the elevation of bone mass by 2G may be due to the up regulation of bone formation.

**Figure 4:**
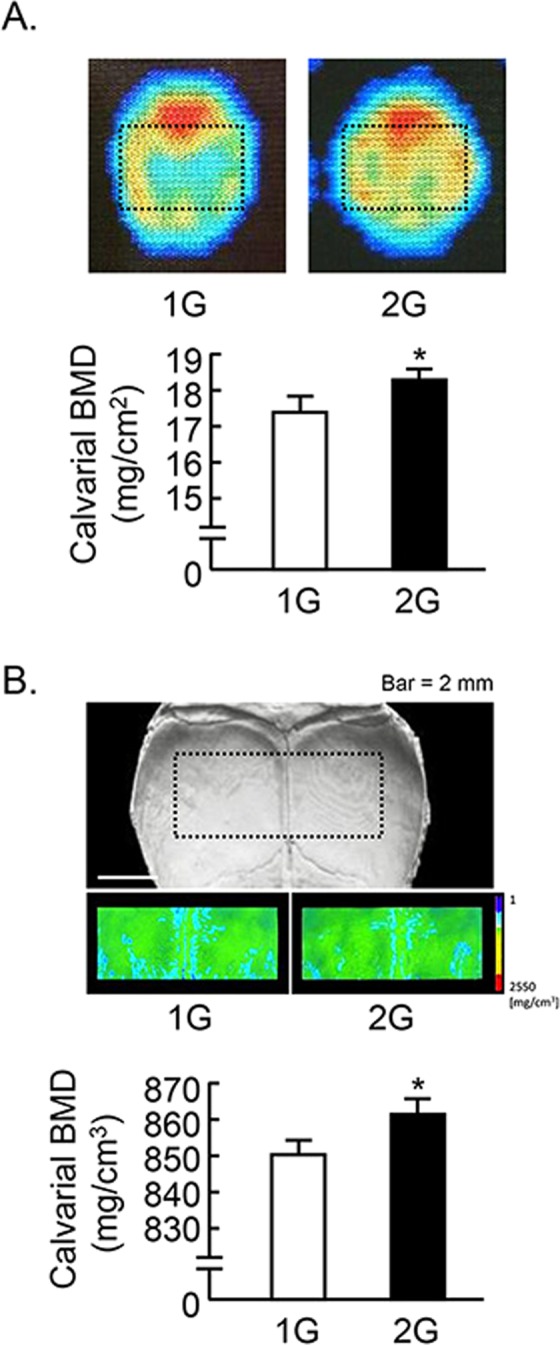
Analysis of calvarial bone mass in 2G mice. (**A**) The BMD images of calvaria were collected by DXA and black dotted square indicated analyzed area (upper images). The calvarial BMD was measured using DXA (lower graph). (**B**) Black dotted square on the μCT images of calvaria indicated the analyzed area, and the color images of analyzed area were reconstructed in μCT (upper images). The calvarial BMD was measured using μCT (lower graph). Data are expressed as the mean ± SEM of 7–8 mice. A significant difference between the two groups was indicated; *P < *0*.*05* vs 1G.

We further analyzed the calvarial bone mass. The analyzed area of calvaria were shown by dotted square (Fig. 4). Calvarial bone mineral density (BMD) measured by DXA was slightly elevated by 2G hypergravity (Fig. 4A). BMD images using μCT were shown in Fig. 4B upper images, and the color images showed the increase in BMD by 2G hypergravity. The calvarial BMD measured by μCT was slightly but significantly elevated by 2G (Fig. 4B, lower graph).

### The bone mass of mouse humerus and tibia in JAXA MHU-2 project

**Figure 5:**
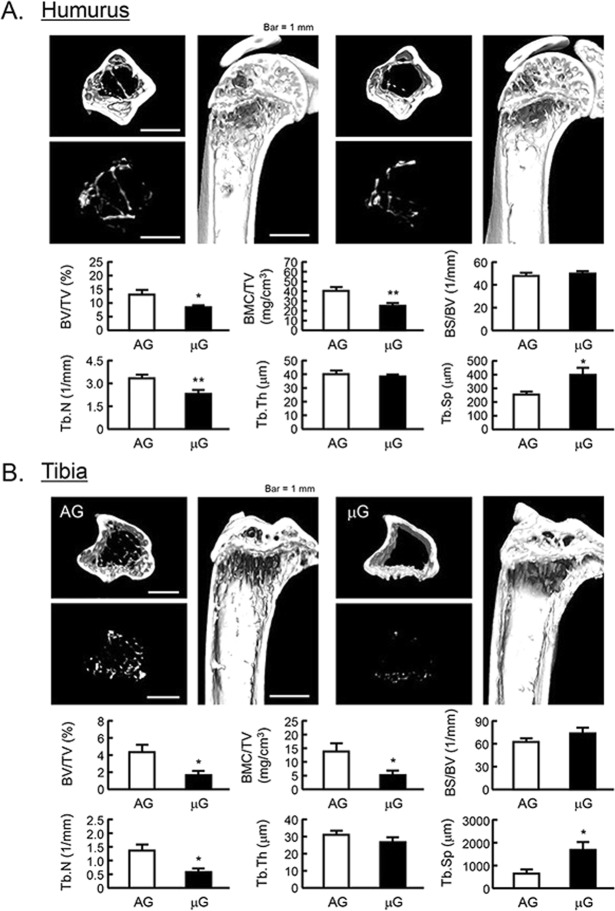
The μCT analysis for the bone mass of humurus and tibia in μG mice. The three-dimensional images (horizontal section, isolated trabecular bone of horizontal section, and vertical section) were shown in upper panels, and bone mass parameters were analyzed by μCT and shown in lower panels in humurus at the proximal region (**A**) and tibia at the proximal region (**B**). The μCT images showed representative bones of 3 mice. The μG means microgravity (spaceflight) and AG means artificial 1G (centrifuged at space). Data are expressed as the mean ± SEM of 3 mice. A significant difference between the two groups was indicated; *P < *0*.*05* and **P < *0*.*01* vs 1G.

Mouse space experiment (MHU-2 project) was conducted by JAXA in 2017. All mice stayed at space for 1 month and successfully returned to earth. Previous report has shown that artificial 1G (AG) at space using centrifuger rescued the space-induced femoral bone mass and muscle atrophy^25^. We analyzed the bone mass of humerus and tibia using μCT in MHU-2 project. The reconstructed μCT images showed that the bone mass was reduced in μG (Fig. 5, upper images). BV/TV, BMC/TV and Tb.N of humerus and femur were significantly decreased, and Tb.Th was tended to decrease in μG mice (Fig. 5, lower graphs). On the other hand, Tb.Sp of these bones was significantly increased, and BS/BV was tended to increase in μG mice (Fig. 5, lower graphs). These data indicated that μG by spaceflight decreased bone mass not only tibia but also humerus and tibia.

## Discussion

In the present study, we newly developed a gondola-type centrifuge with a 1.0 m arm to analyze hypergravity-induced the changes of bone and muscle mass. The data showed that the 2G hypergravity for 2 weeks induced muscle hypertrophy via the upregulation of myogenic genes and the down regulation of muscle-specific ubiquitin ligases and autophagy-related genes. The 2G also elevated the bone mass including humerus, femur, tibia and calvaria by the increased expression of osteogenic genes in mice. We showed that the μG in spaceflight induced the bone loss in both humerus and tibia in mice. This is also first reports to investigate the gravity-induced humerus alteration of bone mass.

Hypergravity has reported to affect various organ tissues not only bone and skeletal muscle. Yoon G *et al*.^26^ have reported hypergravity increased the level of inducible nitric oxide synthase (iNOS), and iNOS attenuated renal hypoxia/reoxygenation injury in the mouse kidney. They also have reported that hypergravity induced endothelial NOS (eNOS) and HIF-1α to protect against hypergravity-induced hypoxia in the mouse heart and liver. In the next study, we are planning to analyze other phenotype in heart, liver, kidney and various tissues.

In the live video imaging of mouse cage in the centrifuge device, mice moved slowly and lay face down on day 1 after centrifugation. However, mice gradually adapted to the 2G hypergravity, and finally walked freely against the 2G in the cage. Under the 2G circumstances, the body weight, food intake and water intake were decreased on day 1 due to exposure to hypergravity, but these reductions gradually restored by the adaptation to the hypergravity circumstances. In the present study, we firstly showed that 2G hypergravity enhanced the mRNA expression of myogenic marker genes (*Myod*, *Myog*, *Myf5*, *Myh1* and *Myh2*), and suppressed the expression of muscle-related autophagy-related genes (*Lc3b*, *Atg5*, *Atg7* and *Atg16l*) in mice. Therefore, we suggest that muscle hypertrophy in 2G results from the increase in myogenesis and the inhibition of muscle degradation *in vivo*. However, further studies are needed to define a key molecule for myogenesis sensor induced by 2G hypergravity.

There are various reports for hypergravity induced osteoblast differentiation and osteoclastic bone resorption *in vitro*^10–18^. It has been reported that 3G hypergravity stimulated bone formation via the enhancement of Runx2 activity in osteoblasts, and 30G hypergravity increased the mRNA expression of *Trap* and *Ctsk* in osteoclasts *in vitro*^14,16,18^. Kawao *et al*.^27^ have reported that 2G centrifugation for 2 weeks significantly elevated the mRNA level of *Osx*, *Alp* and *Col1* in the tibia of mice.

In the present study, we showed that the mRNA expression of the marker genes for osteoblast differentiation, *Bmp2* and *Col1a1* were elevated by 2G in mice. The change of genes for osteoclast differentiation, *Rankl* and *Opg* expression was tend to elevated but not significant. These data suggested that 2G hypergravity leaded to high bone turnover mainly by the increased bone formation, and total bone mass was elevated by 2G in mice. However, molecular mechanisms of 2G hypergravity in bone tissue is still not clear. In the next study, we will try to identify a key molecule of mechanosensing in cells derived from bone using 2G experiments. Our data suggested that 2G hypergravity increased the trabecular bone mass of long bones including humerus, femur and tibia. Remarkably, the increase rate of bone mass by 2G was approximately 2-fold in humerus, 1.3-fold in femur and 1.5-fold in tibia compared with 1G control in mice, indicating that the humerus bone was more sensitive to the hypergravity condition than femoral and tibial bone. In the artificially produced 2G condition, mice tended to fall foreword to any movement that was due to hypergravity and mice was supported the body using forelimb that resulting in excess mechanical stimulation in forelimb. In contrast to the trabecular bone, cortical bone mass was not influenced by 2G hypergravity in all femur, tibia and humerus (data not shown). Since bone formation rate is higher in trabecular bone than in cortical bone, further studies are needed to define the molecular mechanisms induced by 2G in each bone. In the study also revealed that 2G hypergravity also increased calvarial bone mass. It is likely head-linked muscles such as occipitofrontalis muscle might conduct the gravity force to calvaria. Further investigation needs to define the mechanism for increased calvarial bone mass in 2G circumstance. Analysis of video monitoring and foot step of the mice will be clarifying the gravity related mechanism for hypergravity of respective bone.

Under the μG experiments in the space, JAXA conducted mouse space experiments, called as MHU-1 in 2016. JAXA already prepared the artificial 1G centrifuger in space with a unique mouse cage unit on MHU projects^24,25^. In the present study, MHU-2 project in 2017, we analyzed bone mass of humerus and tibia of the mice that was keeping on breeding at space for 34 days. The trabecular bone mass of both humerus and tibia was significantly decreased in μG mice compared with artificial 1G mice. In this report, we measured for the first time the alteration of humerus in μG circumstance. In μCT analysis, both of humerus bone and tibial bone increasing trabecular separation with decreasing bone volume/tissue volume, suggesting that osteoclastic bone resorption was increased in microgravity circumstance. Artificial 1G recovered both of bone volume with increasing trabecular number that thought to be increased bone formation in osteoblasts. In contrast to the trabecular bone, cortical bone mass was not influenced by space μG for 34 days (data not shown). Further studies are needed to compare trabecular and cortical bone in bone-turnover rate in μG condition.

Some of reasoning could be thoughtful for the molecular mechanisms of gravity responding bone and muscle mass alteration. In this study, we firstly observed forelimb humerus have influenced microgravity and artificially produced 1G that recovered bone mass in addition to alteration of bone mass in hindlimb. Further study is needed to observe muscle mass on hindlimb in the space. We currently analyzed tail suspension mice and found that the level of PGE_2_ production was elevated and bone mass was decreased (unpublished data). Since PGE_2_ discovered the inflammatory mediator of bone resorption, the gravity and force may have influenced alteration of bone mass. It is possible that gravity influence PGE_2_ production and the balance of bone resorption and formation in mice. Further investigation is needed to clarify the molecular mechanisms of gravity-regulated bone mass in mice.

In conclusion, 2G centrifugation using a gondola-type centrifugal device with a 1.0 m arm for 14 days that regulates myogenic genes for degradation and formation and bone-related genes. The alteration associated with hyper gravity under 2G increased bone and muscle formation, leading to muscle hypertrophy and increased bone mass. In addition, we found that gravity altered the bone mass of humerus in both circumstances of 2G and μG. We also found that in the 2G circumstances, mice resisted to 2G to move, indicating that exercise with hyper weight loading induce bone and muscle volume, however, the relationship between gravity and exercise has to be examined. Here we suggest that bone and muscle mass are regulated by the gravity with loaded force in both positively and negatively on the ground and in the space.

## Materials and Methods

### Animals and reagents

Five-week-old C57BL/6J male mice were purchased from the Jackson Laboratory (ME, USA) for the space experiments. Twelve mice at 9 weeks old were selected for spaceflight based on body weight and water/food consumptions, and were flown to the ISS by SpaceX-12. Animal protocols were reviewed and approved by the Institutional Animal Care and Use Committee of JAXA (protocol number; 016–018), NASA (protocol number; FLT-17–106) and Exlora BioLabs (EB15-010). Eight- and eleven-week-old C57BL/6J male mice were purchased from the Charles River Labolatories Japan, Inc. (Ibaraki, Japan) for the hypergravity experiment on ground. After the 3 weeks acclimation, animals were set on the JAXA centrifuge and were reared for 2 weeks at 2G. Animals protocols were reviewed and approved by the Institutional Animal Care and Use Committee of JAXA (protocol number; 017–035) and Tokyo University of Agriculture and Technology (protocol number; 30–3). All methods were performed in accordance with the relevant guidelines and regulations.

### 2G hypergravity produced by centrifugation

The 2G environment was applied by centrifugation of custom-made gondola-type rotating box with a 1.0 m arm (Advanced Engineering Service, Tokyo, Japan) (Fig. 1A, left). To obtain 2G, 40 rpm was applied. An animal habitat cage with CRF-1 food (Oriental Yeast Co., Ltd., Tokyo, Japan) and water bottle was set in a box (Fig. 1A, right), and the centrifuge hold maximally 6 rotating boxes. The video camera set in the box monitored behaviors of mice during centrifugation. The centrifuge was placed in an air-conditioned room (24 ± 2 °C) with a 12:12 h light-dark cycle.

### Micro-computed tomography analysis

The volume of calf muscle around the tibia including the triceps surae muscle was measured using micro-computed tomography (μCT) (inspeXio SMX-90CT; Shimadzu, Kyoto, Japan). Bone tissues such as calvaria, humerus, femur and tibia, were collected from mice and fixed in 70% ethanol. The bone mass in the central region of calvaria, the proximal area of humerus and tibia, and the distal area of femur was measured using μCT. Three-dimensional reconstructed images and the bone mass parameters, bone volume/tissue volume (BV/TV), bone mineral content/tissue volume (BMC/TV), trabecular number (Tb.N), trabecular thickness (Tb.Th), bone surface/bone volume (BS/BV), trabecular separation (Tb.Sp), cortical bone mineral density (BMD), cortical thickness and cross sectional area were calculated using TRI-3D-BON software (RATOC System Engineering, Tokyo, Japan).

### Dual-energy X-ray absorptiometry

The bone mineral density (BMD) at the central region of calvaria was measured by dual-energy X-ray absorptiometry (model DCS-600R; Aloka, Tokyo, Japan).

### Quantitative PCR analysis for mouse trabecular bone, and muscles quadriceps

Mouse trabecular bone with bone marrow was collected from tibia. Musculus quadriceps was obtained from right hindlimb and frozen by liquid N_2_. Total RNA from these tissues was isolated using ISOGEN (Nippon Gene Co., Ltd., Toyama, Japan). The cDNA was synthesized from RNA through reverse transcription, and the respective genes was quantified by quantitative-PCR (qPCR) with SsoAdvanced SYBR Green Supermix (Bio-Rad Laboratories Inc., CA, USA). The expression level of target gene was analyzed using ΔΔCq (Cq: quantification cycle) methods. The β-actin, 18S and HPRT were used as normalized genes. The sequences of PCR primers are shown in Table 1.

**Table 1:** Primer sequences for qPCR.

Genes	Forward	Reverse
mouse *Myod*	ccttgctcagctccctca	tgggagttgcattcactgg
mouse *Myog*	cccatggtgcccagtgaa	gcagattgtgggcgtctgta
mouse *Myf5*	tgaatgtaacagccctgtctggtc	cgtgatagataagtctggagctgg
mouse *Myh1*	gagggacagttcatcgatagcaa	gggccaacttgtcatctctcat
mouse *Myh2*	aggcggctgaggagcacgta	gcggcacaagcagcgttgg
mouse *Atrogin1*	cagcttcgtgagcgacctc	ggcagtcgagaagtccagtc
mouse *Murf1*	gtgtgaggtgcctacttgctc	gctcagtcttctgtccttgga
mouse *Lc3b*	aagcagcgccggagctttga	gagctgcaagcgccgtctga
mouse *Atg5*	gtggtttggacgaattccaac	agagctgaacttgatgcaaga
mouse *Atg7*	cacggttcgataatgttcttcc	gaatccttctcgctcgtactg
mouse *Atg16l*	ccaggaggcgtcaagcacgg	tgctgacagctcggacggga
mouse *Bmp2*	gtcgaagctctcccactgac	caggaagctttgggaaacag
mouse *Col1a1*	cttgccagcttccccatcatct	catgggtccttctggtcctcgt
mouse *Osx*	aacttcttctcccgggtgtg	tgaggaagaagcccattcac
mouse *Sost*	gtgtgatgttgggctacgtg	ccaccacaatctctccccta
mouse *Rank*	tgctcctcttcatctctgtggtagtagtgg	tggagtgaagtctgcgccccaccctgctcc
mouse *Rankl*	aggctgggccaagatctcta	gtctgtaggtacgcttcccg
mouse *Opg*	agcaggagtgcaaccgcacc	ttccagcttgcaccacgccg
mouse *Actb*	ccccattgaacatggcattg	acgaccagaggcatacagg
mouse 18 *s*	tcaagaacgaaagtcggagg	ggacatctaagggcatcac
mouse *Hprt*	tcagtcaacgggggacataa	ggggctgtactgcttaaccag

### MHU-2 project

Experimental designed including the mouse habitant cage unit (HCU) and mouse transportation cage unit (TCU) was established as described in previous report^24,25^. Mice were stayed for 34 days with artificial 1G or μG in the space. After spaceflight, all living mice were splashed down in the Pacific Ocean off the coast of California. Mice were stayed for 2 days in TCU on the sea, and TCU was picked and transported to Explora Biolabs in San Diego for analysis.

### Statistical analysis

Data are presented as the means ± SEM. The significance of differences was analyzed using Student’s t-test.

All experiments were performed in accordance with the relevant guidelines and regulations of JAXA and Tokyo University of Agriculture and Technology.
